# Fertility in women of late reproductive age: the role of serum anti-Müllerian hormone (AMH) levels in its assessment

**DOI:** 10.1007/s40618-016-0497-6

**Published:** 2016-06-14

**Authors:** B. Meczekalski, A. Czyzyk, M. Kunicki, A. Podfigurna-Stopa, L. Plociennik, G. Jakiel, M. Maciejewska-Jeske, K. Lukaszuk

**Affiliations:** 1 Department of Gynecological Endocrinology, Poznan University of Medical Sciences, ul. Polna 33, Poznan, Poland; 2 INVICTA Fertility and Reproductive Center, Gdańsk, Poland; 3 INVICTA Fertility and Reproductive Center, Warsaw, Poland; 4 Department of Obstetrics and Gynecological Nursing, Faculty of Health Sciences, Medical University of Gdansk, Gdańsk, Poland; 5 Department of Obstetrics and Gynecology, Center of Postgraduate Education, Warsaw, Poland

**Keywords:** Fertility, Late reproductive age, Anti-Müllerian hormone, Pregnancy

## Abstract

**Introduction:**

Fertility is referred to the capability for having offspring and can be evaluated by fertility rate. Women’s fertility is strictly dependent on individual’s age. The fertility peak occurs in the early 20s, and it starts to decline in the third and fourth decades of life (falling sharply after age 35).

**Aim:**

The aim of this work is to review the available data concerning fertility in women of late reproductive age, especially the role of serum anti-Müllerian hormone (AMH) levels.

**Results:**

There are a lot of factors responsible for decrease of fertility in women of late reproductive age. These factors can be classified as oocyte-dependent (decrease in oocyte quantity and quality) and oocyte-independent (reproductive organs [uterus, oviducts] status and general health). Anti-Müllerian hormone (AMH) is a dimeric glycoprotein of the transforming growth factor-β (TGF-β) superfamily produced directly by the ovarian granulosa cells of secondary, preantral, and early antral follicles. It has been used as an ovarian reserve marker since 2002. Anti-Müllerian hormone seems to be the best endocrine marker for assessing the age-related decline of the ovarian pool in healthy women. Evaluation of AMH’s predictive value in the naturally aging population is important for counseling women about reproductive planning as well as for treatment planning for women experiencing hormone-sensitive gynecological conditions such as endometriosis and fibroids.

**Conclusions:**

AMH can be considered as an indicator of fertility in late reproductive age women and pregnancy outcome in assisted reproductive technology cycles. AMH can strongly predict poor response in the controlled ovarian stimulation.

## Introduction

Fertility can be defined as natural capability for having offspring and measured using fertility rate, i.e., the number of live births per 1000 women. In demographic or socioeconomic context, fertility is measured using total fertility rate index, which is a the total number of children per women during her whole life [1]. It is well known that women’s fertility is strictly dependent on individual’s age and that fertility peak occurs in the early 20s and it starts to decline in the third and fourth decades of life (falling sharply after age 35) [2]. Menopause is understood as a natural cessation of women’s reproductive ability. It typically occurs in women in late 40s and early 50s [3]. Therefore, conceiving a baby by women between older than 35 years old can be regarded as fertility in late reproductive age. It has been estimated that at age 35 and 40, 66 and 44 % women, respectively, will have a conception ending in live birth within 1 year [4].

Smith and Buyalos [5] prepared the statistical analysis which presented the age-dependent decrease in female fertility. This decline in fertility rate is becoming clearly visible at the female age 38 years.

According to these data, the median age at last birth is 40–41 years old across a range of natural fertility populations. Therefore, the start of a “natural infertility” period can be estimated to start about 8 years before menopause.

In this review, we will briefly review available data concerning the causes of decrease of fertility in late reproductive age and possible diagnostic tools for its assessment.

## Causes of decreased fertility

There are a lot of factors responsible for decrease of fertility in women of late reproductive age. These factors can be classified as oocyte-dependent and oocyte-independent [5]. The first group refers to the decreasing number and quality of oocytes with advancing age, and the second is related to reproductive organs (uterus, oviducts) and general health.

## Oocyte-dependent causes

The loss of oocytes is a continuous process that begins just after the establishment of the oocyte pool during fetal life. At approximately 20 weeks’ gestation, the ovaries in the female fetus contain 6–7 million oocytes, but this number falls rapidly. In total, 1–2 million oocytes remain at birth, and only 300,000–500,000 are present at age of 13 at the onset of puberty [6]. In perimenopausal women, we can find only few hundred (750–1000) of oocytes [7]. If we realize that during reproductive period only 500 oocytes can ovulate, it is obvious that vast of those are lost through apoptosis.

Recent studies presented the model to evaluate the decrease in ovarian nongrowing follicle reduction in relation to female aging [8] showing that the rate at which ovarian nongrowing follicles are lost changes smoothly rather than suddenly.

With the loss of ovarian follicular pool, women experience cycle shortening, menstrual irregularity, infertility, sterility and finally menopause. Changes in menstrual regularity are observed approximately 6 years before the beginning of menopause [7, 9].

Child-bearing usually ends 8–0 years before menopause, and this time period is consistent regardless of the age of menopause [9].

Selection of a dominant follicle which occurs earlier than normally and is caused by the shortening of the first phase of menstrual cycle in older women [10]. Broekmans et al. [11] reviewed the contemporary knowledge of the ovarian aging in relation to fertility and menopause.

Age affects not only the size of the oocyte pool but also the quality of germ cells. Farr et al. [12] studied the number of live births in different age groups after transfers of embryos from eggs from donors in comparison with live births after ART cycles using woman’s own eggs. The results showed that the pregnancy rate depends mostly on the age of oocyte donor and the live births rate in ART declines with female age. Even in women of advanced age (up to 60 years old), if the oocyte donor were young (20s to early 30s) the number of life births remains relatively high) [9].

Other proof for decreasing quality of oocytes with age is rate of oocyte aneuploidy. The rate is low for women by up to age 35 (53 % in embryos after 3 days from fertilization), but increases to 74 % at the age of 41–42, and to 93 % after the age of 42 [13].

Oocyte quality declines with age and seems to be, at least partially, dependent on meiotic errors. It has been shown that meiotic spindles are more diffuse in older women [14, 15]. It has been proposed that one of the important mechanisms influencing chromatin division is a function of oocyte mitochondria. This hypothesis is based on findings of abnormal spindles and chromosomal scattering in oocytes deficient in the pyruvate-metabolizing enzyme Pdha1 [16].

Also the oocyte selection process seems to become more aberrant with age. Available data from IVF cycles show that in the selection is less discriminating, which allows maturation of follicles, which in younger women would have undergone atresia [17].

## Oocyte-independent causes

In addition to the changes discussed above related to oocyte quality, the ability to achieve and maintain a pregnancy is also dependent on the uterus. Decidual and placental development and embryo implantation are strictly uterus dependent, but epidemiological data show weak association between maternal age and stillbirth rate [18]. This correlation remains even after correction for potential confounders, including fetal chromosomal abnormalities, multiple pregnancy, obesity, pre-eclampsia, insulin-dependent diabetes and multiple pregnancy [19]. Animal studies allowed identification of some of the factors contributing to this correlation. In mice, older ones showed an impairment of artificially induced decidual response in comparison with the young [20]. There was an observed tendency for compensatory placental hypertrophy in older individuals [21]. Therefore, decidualization and placentation processes may be impaired in women of advanced age.

Recently some data have been published about possible role of cumulus cells function in the process of ovarian aging [22]. Oocyte function is dependent on its microenvironment, and the bidirectional communication between the cumulus cells and the oocyte plays a crucial role. It has been noted that aging is connected with an increase in expression of genes related to angiogenesis in cumulus cells. One of the proposed mechanisms affecting chromosome segregation is hypoxia [25].

Other factors related with advancing women’s age such as uterine pathology, fibroids, endometriosis and endometrial polypus, dysfunctional response of endometrium to hormonal stimulation and finally general health (cardiovascular diseases, metabolic diseases, oncological diseases) can also be considered [23].

## Short summary of available data concerning role of AMH in determination of ovarian reserve and physiological rationale

Anti-Müllerian hormone (AMH) is a dimeric glycoprotein of the transforming growth factor-β (TGF-β) superfamily involved in cell growth and differentiation [24]. It is produced directly by the ovarian granulosa cells of secondary, preantral, and early antral follicles up to 6 mm in diameter, and its secretion ceases as follicles grow into dominance [25].

AMH production was first demonstrated almost 35 years ago by Hutson et al. [26].

This hormone appears in the 36th week of gestation and decreases continuously through puberty. It becomes undetectable when menopause occurs [27]. There are only slight changes in serum AMH level during the menstrual cycle and AMH can be measured at any day of the menstrual cycle. The main physiological role of AMH in the ovary seems to be limited to the inhibition of early stages of follicular development and prevention of the recruitment of a nondominant follicle [28].

Serum AMH levels have been shown to correlate with ovarian reserve (follicular pool) [29]. It has been used as an ovarian reserve marker since 2002 [30]. Ovarian reserve can be defined as the number and quality of follicles in the ovary at any given time. Evaluation of ovarian reserve is recommended for patients who are at risk of decreased or diminished ovarian reserve (DOR). Although the level of AMH is a good predictor of oocyte quantity, it may not provide information about egg quality. Thus, young women with low AMH levels may have a reduced number of oocytes but normal, age-appropriate oocyte quality [31].

In 2003, Fanchin et al. demonstrated that antral follicle count (AFC) was closely related to serum AMH level on the third day of cycle in infertile women. This correlation was shown to be stronger than with other hormonal markers such as inhibin B, E2, and FSH [32].

Many studies have shown that AMH is currently the best available measure of ovarian reserve applicable in a variety of clinical situations, such as infertility treatment (especially IVF), forecasting of reproductive lifespan, ovarian dysfunction (especially polycystic ovary syndrome), and gonadotoxic cancer treatment or ovarian surgery. Moreover, AMH may help to individualize ovarian stimulation protocols, thereby improving the efficiency and safety of IVF [33].

## Evolution of AMH measurements methods

The first AMH assay was described by Hudson et al. [34] in 1990. The first commercially available assays (so-called first-generation assays) were introduced by Diagnostic Systems Laboratories (DSL) and Immunotech Ltd (IOT). Each new assay used different standards and antibodies. The majority of studies showed that the results obtained with the IOT assay had been higher in comparison with the DSL kit. However, there were also some data that had shown their equivalence [35–37].

The ELISA Gen II Assay (Gen II) was introduced by Beckman Coulter in November 2010 aiming to combine and replace the two first-generation methods. In the preliminary studies, Kumar et al. [38] found that the results obtained with this assay had been correlated and higher than those given by DSL. The similar results were shown by Wallace et al. and Nelson and La Marca [39, 40] who found the 40 % higher concentrations obtained by Gen II assay. Surprisingly, the next investigation presented different results in which AMH results obtained with Gen II were found to be 20–40 % lower than those measured using the DSL assay [41].

These discrepancies led to confusion among scientists and clinicians. Many factors, e.g., sample storage conditions (−20 vs. −80 C), storage time prior to assessment of sample (fresh vs. storage of 7 days), and other preanalytical and analytical conditions could lead to different results and finally lack of reliability of methods [41, 42].

The retrospective analysis of 8323 blood samples that compared IOT assay with Gen II found that the correlations of clinical results with the patient AMH level showed large differences depending on the AMH assay used. These varying values of AMH depending on the particular assay used could result in erroneous clinical decisions with regard to stimulation protocols in IVF [42]. Additionally, the letter published in Fertility and Sterility noted that patients with a high risk of ovarian hyperstimulation syndrome (OHSS) should have their AMH tested paying careful attention to the test used in order to avoid applying an unsuitable stimulation protocol [43]. The request for explanation of such discrepancies in results directed to Beckman Coulter remained unanswered.

However, the observed discrepancies resulted in the withdrawal of the Gen II assay from the market in July 2013 (Beckman Coulter UK Urgent Field Safety Notice 20434-3). The next step in development of AMH assay was the introduction of a new modified the Gen II assay (Gen IIm) in August 2013. The novelty of that method was based on preliminary premixing of the calibrator, control, or serum and assay buffer to the ELISA plate instead of a sequential step as in the previous kit [44, 45].

In 2014, Ansh Labs introduced two new AMH ELISAs. They were marketed as an ultra-sensitive Ansh Labs ELISA (Ansh-US) for standard use and a picoAMH (Ansh-pico) assay for use in samples with lower AMH. The Ansh-US assay performance characteristics are similar to the Gen IIm. Additionally, it was demonstrated that Ansh-pico has enhanced sensitivity in comparison with previous methods and could be used when low AMH levels were expected. All above described assays were manual ones [46, 47].

The recent years have brought forth the introduction of totally automatic methods: Elecsys^®^ AMH assay by Roche Diagnostics and Access AMH developed by Beckman Coulter. These automatic methods avoid the influence of handling methods on AMH results. Additionally, the results are obtained faster; they have low inter-laboratory variability and finally have better reproducibility than manual assays [48–51].

## AMH as an indicator of fertility in late reproductive age women, spontaneous pregnancy rate, and pregnancy outcome

As mentioned above, AMH levels have been shown to be age dependent [52]. The decrease in AMH levels that occurs with increased age may be noted before changes in other age-related variables, suggesting serum AMH levels may be the best marker of ovarian aging [53].

However, it should be noted that AMH natural values show large inter-individual variability by age, indicating a wide range of ovarian reserve among the healthy population [54].

Cui et al. [55] studied median AMH levels in Chinese women across different age groups. Their results were as follows: 2.35 ng/mL for ages 20–31 years, 1.58 ng/mL for ages 32–34 years, 1.30 ng/mL for ages 35–37 years, 0.96 ng/mL for ages 38–40 years, 1.05 ng/mL for ages 41–43 years, and 0.67 ng/mL for ages >43. This study shows the significance of serum AMH levels decrease with increasing age.

AMH has emerged as marker of ovarian reserve and possible surrogate measure of reproductive aging. Freeman et al. [56] found that AMH was a stronger predictor of median time to menopause than FSH and inhibin b in late reproductive age women. Similar results were published by Depmann et al. [57].

From this point of view, evaluation of AMH’s predictive value in the naturally aging population is important for counseling women about reproductive planning as well as for treatment planning for women experiencing hormone-sensitive gynecological conditions such as endometriosis and fibroids.

There are a very limited number of studies analyzing fecundability in women of advanced reproductive age who want to conceive naturally. It results from the fact that AMH has been used primarily to assess fertility of women undergoing assisted reproduction procedures. Steiner et al. [58] studied women in their late reproductive life (30–42 years). In this study, reduced fecundability was found in women with very low AMH levels (<0.7 ng/ml). Contrary low AMH in healthy women in their mid-20s did not predict reduced fecundability [59]. Additional studies are required to estimate ability of AMH to predict the chance of spontaneous pregnancy in young and particularly in late reproductive age women.

Freeman et al. [60] analyzed the hypothesis that AMH levels are lower in obese women of late reproductive age in comparison with nonobese women in the same age group, and found it to be true. These findings offer further evidence of the complex relationships between obesity and reproductive hormone levels in women.

Ovarian reserve testing should be performed for women older than 35 years who have not conceived after 6 months of attempting pregnancy and women at higher risk of diminished ovarian reserve. At present, it is ACOG Committee’s opinion that ovarian reserve testing results cannot be extrapolated to predict the likelihood of spontaneous conception (ACOG) [61].

## AMH as an indicator of fertility in late reproductive age women and pregnancy outcome in ART cycles

It is a well-known fact that female fertility is diminished with increasing age. According to some data, the atresia of follicle pool is accelerated in women over 37–38 years. When analyzing IVF results published by Human Fertilization and Embryology Authority (HFEA), the live birth rate for the age range 40–42 was 12.7, 5.1 % for ages 43–44, and 1.5 % for women aged 45 and older [62]. It is well established that the success of IVF procedures depends on many factors among which the most important and independent ones are: age and serum AMH concentration [63–66]. AMH can strongly predict poor response in the controlled ovarian stimulation. Despite presented results, the data still showed that women over 40 years with very low AMH concentrations had a chance of pregnancy [67].

There are some studies showing the possibility of pregnancy and live birth in women with low and extremely low AMH and older than 40 years.

Lee et al. [68] found no clinical pregnancies in women over 40 years who had AMH <0.48 ng/mL. In the study presented by Nelson et al. [69], the small group of 26 women with extremely low AMH concentrations ≤0,15 ng/ml achieved no pregnancy during antagonist or modified natural IVF. The age of women was up to 44 years.

In contrast, Weghofer et al. [70] presented a retrospective study showing 1.7 % deliveries per cycle for patients 42 years of age. The cutoff value for AMH was set out in this study on ≤0.4 ng/ml. The pregnancy chances were also presented by Łukaszuk et al. [71] who showed that among women with low AMH (≤0.4 ng/ml) who had undergone long agonist treatment had 5.6 % clinical pregnancy and 2.8 % live birth per patient in group of women >39 years.

Among 48 cycles of women aged >42 with AMH levels of ≤0.2 ng/ml, no pregnancies were observed in Kedem study. In the same study, 3 ongoing pregnancies out of 192 cycles (1.6 %) were recorded for women with AMH levels in the range of 0.2–1.0 ng/ml [72]. Bhide et al. [73] presented data showing reasonable pregnancy chances in women over 40 years at the rate of 5/54 (9.3 %) when AMH was below 10.28 pmol/l. Additionally, no cutoff AMH was established below which pregnancy was excluded. Reicheman et al. [74] found 3.9 % clinical pregnancy rate in women with AMH <0.17 ng/ml and >42 years. Seifer et al. [75] conducted the largest study in this area analyzing AMH concentrations in over 5000 cycles from the Society for Assisted Reproductive Technology Clinic Outcome Reporting System Database for 2012–2013. According to their data, the cycles of women with the AMH concentration lower than 0.16 ng/mL had a greatest risk of cancelation.

The different results in all above studies could be partially explained by different assays applied in different years [75]. In addition, even the similar kits achieved higher or lower values during the same time. Our unpublished data also suggested that AMH could vary even during the same menstrual cycle. Thus, the simple comparison of the results from the various studies may be seriously flawed. It seems that nowadays only the new totally automated assays such as Roche Elecsys could provide data that could give comparable results.

Based on available data, the pregnancy chances for women of advanced reproductive age with low AMH and especially for those over 42 years old are very low but still the available methods fail to dependably predict who will become pregnant.

## Conclusions

Fertility is the leading attribute of female life. It is strictly dependent on individual’s age. Nowadays, there is strong tendency to postpone entry to motherhood. Therefore, oocyte factors (decrease in oocyte quantity and quality) are the main causes responsible for decrease of fertility in women of late reproductive age. Anti-Müllerian (AMH) hormone seems to be the best endocrine marker for assessing the age-related decline of the ovarian pool in healthy women. AMH can be considered as an indicator of fertility in late reproductive age women and pregnancy outcome in assisted reproductive technology (ART) cycles. AMH can strongly predict poor response in the controlled ovarian stimulation.
